# Reflecting on Cancer Pain as Constant Acute Pain, not Chronic Pain. ‘Known Knowns, Known Unknowns, Unknown Unknowns’

**DOI:** 10.1007/s11912-025-01642-w

**Published:** 2025-04-03

**Authors:** Dympna Waldron, Kirk Levins, David Murphy, Michael McCarthy, David Gorey, Karen Ryan, Eileen Mannion, Bairbre Mc Nicholas, Doiminic Ó Brannagáin, Leona Reilly, Laura Gaffney, Beth Molony, Mary Healy, Jack Molony, Anthony Dickenson

**Affiliations:** 1 School of Medicine, National University of Galway, Galway, Ireland; 2 Department of Palliative Medicine, Galway University Hospital (GUH), Saolta Hospitals Group (SHG), Galway, Ireland; 3 Department of Pain Medicine, St Vincent’s Hospital, Dublin, Ireland; 4 Department of Oncology, GUH, SHG, Galway, Ireland; 5 Department of Nephrology, GUH, SHG, Galway, Ireland; 6 Department of Palliative Medicine, Mater Hospital, Dublin, Ireland; 7 Palliative Medicine, National University of Ireland Dublin, Dublin, Ireland; 8 Department of Intensive Care Medicine, GUH, SHG, Galway, Ireland; 9 Department of Palliative Medicine, Our Lady of Lourdes Hospital, Drogheda, Ireland; 10 Royal College of Surgeons in Ireland, Dublin, Ireland; 11 Department of Post Graduate Medicine, University of Limerick, Limerick, Ireland; 12 Clinical Nurse Specialist, Our Lady’s Hospice & Care Services, Dublin, Ireland; 13 General Practitioner Training Scheme, Dublin North Side, Dublin, Ireland; 14 Neuroscience, Physiology and Pharmacology, University College London, London, UK

**Keywords:** Opioid Responsive, Acute pain, Cancer pain, Chronic pain, Nociplastic pain, Endogenous Opioids, Immune System, Fight-or-flight, Tolerance, Opioid-induced Hyperalgesia, Delirium

## Abstract

**Purpose of Review:**

to explore a paradigm shift in the definition of opioid-responsive cancer pain in this hypothesis-driven review. Opioid-responsive cancer pain may be misplaced within the definition of chronic pain, chronic pain takes three months to establish, early effective control is worthwhile to achieve.

**Recent:**

findings, from a bench-to-bed perspective, debates the interpretation of results supporting the hypothesis that opioid-responsive cancer pain could remain ‘constant acute pain’, with explanations, best solutions, for tolerance and/or addiction, in cancer patients compared to those with chronic pain from other conditions.

**Summary:**

Unraveling the unique apparent properties of opioid-responsive cancer pain empowers knowledge of the process by which acute pain may have the potential to remain acute in nature and not transition into chronic pain. Findings outlined defend the hypothesis of probable sustained acute nature of opioid-responsive cancer pain, importance of early, sustained pain control, opioid reduction and further exploration of this hypothesis in clinical practice.

*“What we know is a drop**** what we don’t know is an ocean.”*Issac Newton** [1].

## Introduction

Patient fears of intractable cancer pain are a problem encountered by many clinicians worldwide. Paice et al. [2], reported that *“chronic cancer pain is a serious complication of malignancy or its treatment*. *Currently*,* no comprehensive*,* universally accepted cancer pain classification exists.”* Despite over three decades since the publication of the World Health Organization (WHO) cancer pain guidelines [3], there has been a substantial body of evidence indicating that the treatment of cancer pain is suboptimal [4]. A clearer understanding of the nature of cancer pain is needed if we are to optimize its management [2]. This paper highlights the evidence that opioid-responsive cancer pain (ORCP) serves as a warning of disease for many patients and, even with effective pain management, continues to behave like acute pain regardless of the time elapsed since its onset. Combining current scientific evidence with clinical insights, this work explores the importance of redefining ORCP, drawing on concepts from the definition of chronic pain.

A clear definition is crucial for consistent research implementation and interpretation, ultimately guiding clinical practice. Chronic pain is typically defined as *“pain that persists beyond normal healing time* [5], *and hence lacks the acute warning function of physiological nociception.”* [6] It is generally considered chronic if it lasts or recurs for more than 3 to 6 months. Recently, suggestions to redefine chronic pain as a ‘nociplastic pain syndrome’—where pain itself becomes the disease—have proven valuable for both cancer and non-cancer patient contexts [7]. 

Nociplastic pain is defined by the IASP as [7], *“pain that arises from altered nociception despite no clear evidence of actual or threatened tissue damage causing the activation of peripheral nociceptors or evidence for disease or lesion of the somatosensory system causing the pain.”* These criteria replaced the 2014 clinical criteria for predominant central sensitization (CS) pain and accounted for clinicians’ need to identify (early) and correctly classify patients having chronic pain according to the pain phenotype [8, 9], can explain why they suffer from pain in the absence of a clear origin of nociceptive input or in the absence of enough tissue damage to explain the experienced pain severity, disability and other symptoms [10, 11]. For clinical purposes, CS is defined as an amplification of neural signaling within the central nervous system that elicits pain hypersensitivity [12]. The 2021 IASP clinical criteria for nociplastic pain are important steps towards precision pain medicine [7], yet studies examining the clinimetric and psychometric properties of the criteria are urgently needed [12]. 

In 2015 the International Association for the Study of Pain (IASP) classified and formally recognized pain as a disease for the first time, including cancer pain as one of seven categories of chronic painful conditions. Asthana et al. [13], discuss recent opioid use guidelines, emphasizing that risk-benefit assessments and treatment strategies for cancer patients should be based on evidence specific to this population, rather than simply extrapolated from chronic pain studies. Wong and Cheung [14], discuss the factors that affect the benefits and risks of opioid use in cancer patients and the non-cancer population are quite different.

Santoni et al. [15], asks *“is cancer pain different”?* Blalock and Smith [16], discuss the lack of correlation between immunological and pain research until recently with immune cells now defined as circulating neurons and the immune system as the “sixth sense”, cancer pain initially considered to be dependent on mechanical stimulation was shown to be dependent on the molecular mechanisms of neuroinflammation. Unlike acute pain, chronic pain has no clear physiological benefit as its perpetuation implies evolutive alterations of neuronal plasticity [10]. Donnelly et al. [17], state that *“Neuroimmunology has demonstrated that the “primum movens” of transformation from acute to chronic pain*,* is neuroinflammation*,* which involves a cross-talk between damage sensors (nociceptors of sensory neurons) and immune cells (neuro-immune network)*,* with some of the receptors sensing danger signals common to both immune cells and sensory neurons.”* The response to danger signals that is essential for returning to homeostasis in the short time of acute pain, when it persists, makes chronic pain maladaptive, by sensitizing the neuronal structures of input–output and lowering the pain threshold. Neuroinflammation brings a new unifying vision of chronic pain emerging, the real malignancy of pain is mainly due to the extent of neuroinflammation and neuroimmune dialogue that determines a disease within the disease [15]. 

We believe ORCP is distinct, even though evidence shows nocioplasticity in certain cancer pains, such as cancer bone pain [18], which, despite some opioid responsiveness, is a complex mixed-pain condition requiring multiple management approaches. Chronic pain is generally defined as pain persisting for more than three months beyond the initial insult (e.g., trauma, infection, inflammation), due to an ongoing cause, incomplete resolution, pain presence after apparent healing, or even pain without a clear cause (dolore sine materia) [19–23]. Unlike acute pain, chronic pain lacks a clear physiological benefit, and its persistence can involve evolutionary changes to neuronal plasticity [10]. 

In cancer, pain initially serves as an alert to the presence of disease [2, 9]. In chronic pain, there has been much research over decades leading to more recently thinking that, the pain itself can evolve into the disease [8, 9, 12, 24–31]. Therefore, understanding whether cancer pain functions as acute or chronic pain is a crucial area for exploration. This paper arrives at a timely moment, amid global concerns about opioid addiction [32, 33], aiming to spark discussion about one specific type of cancer pain—opioid-responsive cancer pain (ORCP). The objective of this paper is to propose hypothesis-driven research to build new foundations for understanding ORCP. Key questions include: Are opioids safe as well as effective analgesics for ORCP? If effective, could early and sustained relief prevent the pain from progressing to a chronic or nociplastic state? And do patients with ORCP deserve guidelines that differ from those used for chronic pain management? Based on current discussions and clinical experience, careful opioid titration—and, when needed, de-escalation—often relieves ORCP within hours or days. Many patients achieve stable pain relief, based on the definition that pain is what the patient says it is, though some may develop tolerance [34–37] or opioid-induced hyperalgesia (OIH) [38, 39], conditions that can also be managed effectively, as discussed later in the paper [34–39]. 

Dubin and Patapoutia [40] describe an overview of nociceptors. Hanks [41], defines ORCP as, “*Cancer pain that generally responds in a predictable way to analgesic drugs and drug therapy is the mainstay of treatment*,* successfully controlling pain in 70–90% of patients. The two major problem areas are pain associated with nerve damage*,* and ‘incident” (movement-related) bone pain. Nerve damage pain tends not to respond well to treatment with opioids.”* Marcadante [42], defines ORCP as, “*The degree of analgesia obtained following dose escalation to an endpoint determined by either analgesia or intolerable adverse effects. It is a continuum of response rather than a quantal*,* yes or no*,* phenomenon*,* consistent with the inter-individual variability that characterizes opioid analgesia*,* and the occurrence of dose-dependent effects”*. Interpreting this quote explains the complexities of treating ORCP, ‘Responsivity’ is one component of treating the pain, the continuum being reaching pain control and also involves alterations as cancer progresses or reduces.

There are two subtypes of nociceptive cancer pain: visceral cancer pain (VCP) and a component of somatic cancer pain (SCP). Opioids in general control nociceptive pain [41, 42]. The accepted definition of VCP being, “*pain produced by the stimulation of nociceptors within internal organs*,* that is often poorly localized and can be described as constant and sharp*. *Pain may be acute or chronic*,* depending on its onset and duration.”* [9, 43] Sikandar and Dickenson [44], describe, *“This diffuse nature and difficulty in locating visceral pain is due to a low density of visceral sensory innervation and extensive divergence of visceral input within the CNS.”* SCP “*is easily localized*,* stabbing or boring in character*,* and movement-dependent”* [28, 29] Fallon et al. [45], describe, *“Nociceptive: caused by ongoing tissue damage*,* either somatic (such as bone pain) or visceral (such as gut or hepatic pain);”*

Other types of cancer pain, i.e. somatic non-nociceptive, neuropathic, and treatment effect pains are beyond the scope of this hypothesis. Classification and defense of the hypothesis put forward is helped by adhering to the category of fully opioid responsive pain. This does not mean that the other categories of cancer pain cannot be dealt with, but for the purpose of this paper a distinct and regularly encountered category of pain that is opioid responsive helps to elucidate a clear discussion.

The authors debate that ORCP should be placed within its own category, akin to the acute pain category. Considering the usual ‘acute’, i.e., postoperative/intensive care (ICU) patients, pain improves, ORCP, we believe, warrants its own distinct category as *‘constant acute pain’* as the nature of the underlying cancer usually requires dynamic opioid requirement. Basbaum et al. [19], suggests due to persistence of the causing event, lack of resolution of pain, as its perpetuation implies evolutive alterations of neuronal plasticity. ORCP should be controlled early, and relief sustained, if possible, to prevent it being protracted.

The clinical implications of ORCP are explored below, including ORCP potentially being the functional antagonist to the side effects of opioids, opioids and sepsis; delirium; tolerance; opioid-induced hyperalgesia; addiction; respiratory effects; and safe management of opioids throughout the cancer trajectory. (Table 1.)

**Table 1:** Debate on known knowns, known, unknowns and unknown, unknowns

• Opioid responsive cancer pain often alerts to disease and appears to remain opioid responsive, main message, gaining control of the pain early may avoid pain transitioning into chronic nocicplastic pain after three months. Timing of pain control maybe key. • ORCP should be classified akin to the postoperative/ICU pain category, but with the caveat ‘constant’. Tolerance/OIH responding generally to intervention, [140] which should be initiated early and repeatedly (as required) throughout cancer disease trajectory.[37–39, 71, 140] • In chronic nociplastic pain, pain becomes the disease. [6–13, 91]
• Hypothesis of endogenous opioid production pre-sepsis warrants opioid reduction which could aid diagnosis of sepsis and reduce incidence of delirium. Hypothesis of endogenous opioid production during *‘fight-or-flight’* pre-sepsis and during-sepsis warrants opioid reduction but also recalibration back to pre-sepsis baseline dose post-sepsis for ORCP suggesting a lack of tolerance for this patient population.[56–60, 63] Adherence to this hypothesis could reduce incidence of protracted delirium.[63–66]
• A separate ‘red flag’ sepsis scoring system for patients on opioids, plus naloxone during sepsis warrants attention.
• The *‘image’* of WHO stepladder should reflect ‘*step-up*,* step-down’* in tandem with the clinical trajectory of cancer disease. • Examining the unique properties of *‘chronic nociplastic pain’* versus controlled *‘ORCP’* in Cluster Clinical Trials, inclusive of markers of *‘fight-or-flight’*, miRNA and fMRI studies of brain centers could tease out differences between *’acute’* versus *‘chronic’* pain.
• Palliative Medicine/Pain Specialists, specialists with extensive knowledge of opioids, should engage early in cancer diagnosis to ensure continuity of care for ORCP. To facilitate this a debate would be helpful concerning the earlier involvement of Palliative Medical Specialists to encompass the WHO recent directive.[145] Palliative Medicine/Nursing/Supportive Care in Cancer Centers and/or educate the public of the value of early involvement of the specialty to further reduce fears for the patient and oncologist to involve the *‘Gatekeepers’* of cancer symptom control promptly.[144]

## What is the Evidence to Support that Opioid Responsive Cancer Pain Remains Acute in Nature?

### What is Known: Opioid Responsive Cancer Pain Alerts Patients to Disease

The incidence of chronic pain worldwide is 20–25%, making it a serious public health problem [46]. Deregulation of microRNAs (miRNAs) may act as a master switch of the genome, causing maladaptive plasticity and neuronal plasticity, leading to chronic pain [26]. Clinical experience shows that cancer patients develop tolerance and opioid-induced hyperalgesia (OIH) [34–39]. This cohort of patients appear to respond to appropriate interventions expanded on below in section on tolerance/OIH. Their pain can be controlled throughout most of their disease trajectory, despite the time from initiation of their pain, why? There is evidence that cancer patients do proceed to nociplastic pain while others may not, why [47]? 

It is difficult to render a patient with chronic nociplastic pain pain-free. Mao [48], states *“Chronic pain is therefore not simply a chronological extension of acute pain and requires different diagnostic approaches and management strategies.”* Melzack and Wall’s theory of neuromodulation [24] and Markenson [49], quotes on, *“(1) the transduction of tissue injury or disease signals (nociception and nociceptive receptors); (2) the transmission of signals rostrally to the thalamus and higher nervous system centers (involving perception of the quality*,* location*,* and intensity of noxious signals); and (3) the modulation of ascending sensory messages at all levels (periphery*,* spinal cord*,* and higher centers)”.* This alteration in brain perception/functioning referred to as *Central Sensitization (CS) creates* an environment where *‘pain becomes the disease’*. Despite the timing from initiation of ORCP, patients can be addressed with the right doses of opioids and/or agents to treat tolerance/OIH. For many patients ORCP alerts a person to see their doctor for diagnosis. Effective treatment is given, with the caveat that if tolerance and/or OIH develop, additional measures require enactment as outlined in section below.

Nijs et al. [50, 51], quote, *“the knowledge regarding CS has revealed a paradigm shift in the understanding and management of chronic pain that allows clinicians to think beyond muscles and joints and to account for the role of pain modulation in the central nervous system.”* CS causes alterations in brain centres involved in acute pain and chronic pain, therefore there is a potential with Functional Magnetic Resolution Imaging (fMRI) to differentiate both pain states [31, 52, 53]. This could be one way to research if ORCP does stay acute in nature if control achieved early?

### What is Known, Unknown, Opioids and the Immune System

It is known that opioids produce immunomodulation in both humans and experimental animals; however, there is a need for systematic studies of the immunomodulatory effects of classic opioids to provide firm evidence for the site(s) of action to aid in evidence-based opioid use. Al-Hashimi et al. [54], described “*more questions than answers.*” Additionally, not all opioids induce the same immunomodulation, i.e., buprenorphine, unlike the conventional opioid’s morphine sulfate and fentanyl, does not appear to cause immunosuppression [55]. 

Some of the effects mediated by exogenous opioids are due to the release of endogenous opioid peptides with antinociceptive effects [56]. Additionally, endogenous opioid signaling in the anterior cingulate cortex (ACC) appears to be both necessary and sufficient for relieving pain aversiveness, and in recent chronic pain studies, the role of harnessing endogenous opioids has been examined [57, 58]. Banerjee et al. [59], noted that *“while the systemic analgesic response to morphine undergoes tolerance with chronic use*,* the effects of morphine on immune cells do not”.*

In clinical practice, there is a caveat regarding the interaction between external and endogenous opioids that warrants exploration, i.e., what happens when infection/sepsis is evolving in patients on opioids for ORCP? The initial idea that ORCP may remain acute in nature occurred in early 2000s while setting up the first Specialist Palliative Medical service, single handedly for the West of Ireland, occasionally cancer patients stable on opioids the previous day, presented to Emergency departments in the three major Hospitals our service covered with moderate to severe opioid toxicity. Naturally, it could be considered the patient had received too much opioid. But our service knew through community visits, this was not the case. Then it became clear that many of these patients went on to develop sepsis that was not obvious at presentation. On completing a retrospective review, the thinking became that patients in the prodromal phase of sepsis could be producing endogenous opioids, as ‘fight-or-flight’ response that rendered some or all their external opioids redundant, thereby presenting as opioid toxic [60]. Less than 10% of this patient population did not have cancer. Table 2 shows the timing from opioid toxicity presentation to clear diagnosis of sepsis and the recalibration back towards their pre-sepsis baseline doses after medically effective treatment of sepsis.

On review of all major Sepsis scoring systems none of them mention Opioids as physiological parameters. Inclusion of all patients, on opioids, admitted to an acute hospital may help towards an earlier detection of sepsis, especially for cancer patients who may be on chemotherapy and/or immunotherapy [61, 62]. 

If indeed, the ORCP had become nocicplastic, the expectation could be that the extra endogenous opioid effect would not create quite marked opioid toxicity that on occasions required use of naloxone. Also, it became apparent that the patients with ORCP required recalibration back to, or near, their pre-sepsis opioid dose after treatment of sepsis, which is classified as the *‘recalibration phase’*. Both these factors appeared in line with ORCP remaining acute in nature and not always developing tolerance. Waldron et al. [60], proposed that early signs of opioid toxicity should be captured in a sepsis scoring system for all patients on opioids as it is an additional *‘warning score’* of sepsis.

**Table 2:** Opioids and sepsis; endogenous morphine?

A retrospective study was performed on all patients referred to palliative care in a cancer center, over a 3-month period. The aim was to test the perceived hypothesis that opioid toxicity may herald imminent sepsis, and that the toxicity is potentially related to enhanced endogenous opioid produced in the prodromal phase of sepsis.		
•	150 patient charts studied. 14 nonmalignant. Age, years; mean 60. 60% Male, 40% Female. 88% on regular opioids. 53 patients on regular opioids developed unexpected opioid toxicity.	
*N* = 53		
•	***Presenting feature of opioid toxicity***: 100% had myoclonus. 61% hallucinations and other CNS side effects. 49% sweating. 44% pin-point pupils. 31% hypotension. 29% decreased respiratory rate.	74% had normal white cell count. (0 neutropenic) 74% displayed little clear clinical signs of sepsis at presentation with opioid toxicity. 100% ultimately developed sepsis. ***Time from opioid toxicity to sepsis diagnosis***: 63% within 24 h, 26% within 36 h 11% within 48 h.
•	***Opioid reduction at presentation of toxicity***: 40% of patients, opioids reduced by 50%. 40% of patients, opioids reduced by 100%.	***Use of Naloxone***: Occasional naloxone low-dose subcutaneous (SC) or intravenous route (IV) if respiration rate below 10/minute, i.e., 100 micrograms (MCGS) until patient settled and analgesia not reversed.
•	***Two weeks post effective treatment of sepsis; % opioid requirement reverted near pre-sepsis dose***: 35% to baseline dose. 54–30% off baseline dose. Minimal opioid tolerance seen. This feature defined as the *‘recalibration phase’*. 55% discharged; 35% still hospitalized; 10% RIP.	
•	***Novel policy instigated locally following this audit***: Patients with ORCP, developing unexpected opioid toxicity ***‘check for and presume imminent sepsis’.*** The hypothesis proposed; the body may be producing additional endogenous opioids in preparation for *‘Fight-or-Flight’* of imminent sepsis, hence *‘exogenous opioids’* are temporarily redundant. Limitation, this is a retrospective study.	

Glattard et al. [63], years later, reported that endogenous morphine is secreted from human neutrophils after lipopolysaccharide (LPS)-induced IL-8 stimulation in patients who are septic and not on opioids. If indeed endogenous opioids are increased, these findings reinforced the findings in our work and this patient group were not on opioids. Together, both studies propose the potential for benefit of naloxone use for patients with hypotension/ileus/delirium both pre-sepsis and during sepsis for patients on/or not on opioids to increase the medical reversibility of patients with life-threatening sepsis.

### Known Unknown: Opioids and Delirium, Clinical Application

The commonly accepted finding in patients with hypoactive delirium is a decrease in acetylcholine (ACh) and increased dopamine (DA). Treatment of delirium involves removing the offending cause [64, 65]. In Palliative Medicine the incidence ranges from 28 to 88% depending on the stage of the illness, with a higher number occurring at End-of-Life (EoL) [64]. Increased DA could reflect extra/redundant opioids, as could lowered Ach as Osman et al. reported that morphine delivered to the basal forebrain causes irreversible inhibition of ACh release in the prefrontal cortex [66]. Therefore, sepsis should be considered as causative in delirium and lowering opioids maybe appropriate at all stages of disease.

### What is a Known, Unknown: Opioids and Addiction

While the mechanisms underlying opioid addiction are complex and still intensely debated, one consistent remains, dopamine (DA) neurotransmission is incontrovertibly involved in the development and maintenance of the compulsion to abuse drugs [67]. Navratilova et al. [68], supported *“the concept that the rewarding effect of pain relief requires opioid signaling in the anterior cingulate cortex*,* activation of midbrain dopamine neurons and release of dopamine from the nucleus accumbens.”* The key to all recent research on gamma-aminobutyric acid (GABA) and DA interactions is that chronic pain leads to a hypodopaminergic state that impairs motivated behavior [69]. GABA is known as the major *‘brake’* of the brain, with pain stimulating the production of GABA, thus lowering DA [70]. 

Enna and McCarson [70], suggest, *“the antinociceptive responses to GABA agonists*,* vary as a function of the duration and intensity of a painful stimulus and of drug therapy. Such findings may facilitate the identification of pain syndromes that are particularly responsive to manipulation of GABAergic transmission… is consistent with the notion that manipulation of this transmitter system may be of clinical benefit in the treatment of acute*,* inflammatory*,* and neuropathic pain.”* Again, the study of ORCP as a constant acute pain and/or attempting to keep this type of pain acute could render benefits in our understanding of addiction in cancer.

### What are the Known, Unknowns? The WHO Stepladder and Pain Control in the Context of Cancer Pain Remaining Acute

It is essential that attending physicians categorize and define the pain for each patient. If the pain associated with cancer is ‘Functional’ and if left untreated has little benefit for the patient; therefore, intense attention to the details of the sources of pain is essential [71]. There is a concern that mixing ‘Psychological’ pain, and Functional’ pain, is potentially misleading [71, 72]. Treating Functional’ pain fully, without compromising the patient, creates space for patients to deal with intense psychological suffering with professional support. Both maybe present, but ‘Functional’ pain effectively controlled could help patients manage ‘Psychological’ pain more effectively? Gomes-Ferraz et al. [73], say ***“****the qualitative synthesis of the results demonstrates that most of the evaluated studies have a mixed nature; there are significant methodological differences among them and a low level of evidence in studies relating to the subject of pain evaluation in palliative care.”* The importance of both ‘Pains’ are vital, but using the ‘Psychological’ pain to explain the lack of effective control of ‘Functional’ pain is an understandable concern.

To achieve good pain control in ORCP, individualized pain control is required and should be delivered by experts with access to follow-up around the clock, especially in cancer centers [74]. Each patient is their own control, pain is what the patient says it is (*n* = 1) and each patient is empowered by the attending team to control their own pain, by reporting it. When patients report pain, the appropriate analgesic is administered, the response is observed, and dose titration is performed based on reported pain and the effect of the analgesic given. Care needs to be given to opioid toxicity and/or a lack of response to opioid given [36]. 

As pain is subjective and can increase or decrease, relying on outcome measures of pain with a ceiling effect alone could be misleading, i.e., 0–10 [75]. Previous ORCP, deemed 10–10 could in future reports due to disease escalation become worse than the previous 10–10, how can this be captured? Addition of validated subjective outcome measures of symptoms and quality of life may add value in this scenario [75–77]. 

Patients’ view of their pain can also recalibrate over time based on memory effects and life events. This recalibration, known as ‘Response Shift’ (RS), suggests that if we measure a subjective symptom, such as pain, using an outcome measure without incorporating RS, we may not capture true improvement/deterioration [78, 79]. Until we have pain measurement tools that incorporate RSs with scientific reliability/validity, patients’ words are the best outcome measure. With ORCP defined as what the patient says it is, McInerney et al. [75], reported that physician knowledge of patient-nominated symptom (PNS) and quality of life (QoL) information provided as a graphical ‘clinical tool’ to physicians observed a highly significant improvement in symptom-bother interference with Quality of Life (47%) improved difference in the active group versus the control group [75]. 

**Fig. 1:**
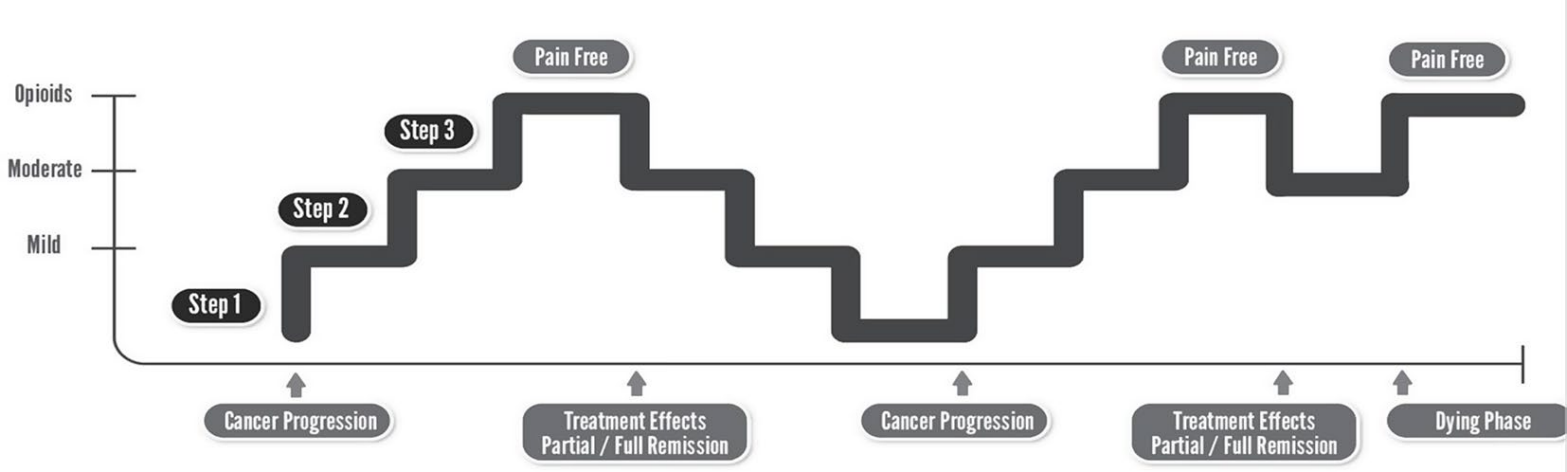
Proposed WHO Stepladder for ORCP reflecting *‘step-up*,* step-down’*

Hypothetically, the average opioid dose at the EoL could potentially be lower if the WHO Stepladder was revised to reflect ‘*Step-up*,* Step-down’* in tandem with cancer disease progression and treatment-induced remission [80]. The image needs to incorporate the disease trajectory we see clinically: (Fig. 1) tolerance, OIH, addiction, opioid side effects, and lowered testosterone in males [81, 82] are potentially lessened if a downward trend is observed. This suggestion is to aid dynamic alterations in opioids over the disease trajectory of cancer, based on the original rationale of WHO to give a simple visual guide world-wide as guidance [3]. It is not to suggest this practice is not happening in other Specialists Centers but centers that may not all have Specialists in Opioid management could be aided by the simple visual image of ‘*Step-up*,* Step-down’*.

### What are the Knowns, Knowns; Respiratory Depression and Opioids

In the postoperative setting described by Pattinson [83], “*modeling has successfully explained pharmacodynamic and pharmacokinetic interactions between CO*_*2*_* and opioids on breathing. With a gradual increase in opioid levels*,* for example*,* with a constant rate infusion*,* progressive respiratory depression causes gradual hypercapnia*,* which contributes to the maintenance of respiration. On the other hand*,* a fast rise in opioid receptor occupancy resulting from an* IV *bolus would lead to apnea until the PaCO*_*2*_* increases to its steady-state value.* He extrapolates from this, opioids with slower binding to receptor (e.g., morphine) may be safer for opioid initiation than opioids that bind more quickly (e.g., alfentanil and remifentanil) even if equianalgesic doses given [83]. All opioids carry concerns of respiratory depression, but fentanyl is usually initiated with ventilatory backup.

The meaning of the above findings for cancer patients with ORCP; initiation of treatment with PO and/or SC morphine with a slower onset of action (up to twenty minutes), which means more time for the intricate maintenance of respiration to occur than the fast-acting lipophilic fentanyl. Learning this caveat from postoperative opioid use maybe helpful for doctors addressing ORCP for initiating opioid treatment in an opioid naïve patient.

### What is Known, Unknown: Tolerance Versus Opioid-Induced Hyperalgesia

Opioid tolerance is characterized by a reduced responsiveness to an opioid agonist such as morphine and is usually manifested by the need to use increasing doses to achieve the desired effect. Opioid rotation (OR) to minimize tolerance and eliminate morphine metabolites has been clinical practice in the maintenance of control of ORCP throughout cancer disease trajectory since it was first alerted to in the 1990s [35, 36]. Knowledge of the potency of each opioid to one another is helpful in the initiation of OR. According to Fine and Portenoy [37], “*Clinically relevant estimates of relative analgesic potency have been codified on the “equianalgesic dose table*,*” which has been used with little modification for more than 40 years”.*

Lee et al. [38], define OIH as, *“Opioid-induced hyperalgesia (OIH) is defined as a state of nociceptive sensitization caused by exposure to opioids. The condition is characterized by a paradoxical response whereby a patient receiving opioids for the treatment of pain could actually become more sensitive to certain painful stimuli.”* There is a problem concerning the dichotomy between OIH and opioid tolerance, although the preclinical and clinical evidence dissociating these two distinct phenomena is mounting [38, 39]. Falk et al. [71] stated, *“While morphine remains the ‘gold-standard’ analgesic*,* the chronic consumption of opioids does not only produce analgesia but can be associated with worsening paradoxical pain sensations; this is the so-called phenomenon of opioid-induced hyperalgesia (OIH).”* Data detailing changes in the central glutaminergic system, spinal dynorphin content and descending facilitations provide a compelling dossier of changes to the neuronal mechanisms that underlie this particular pain state. While the endogenous opioid system is not believed to modulate at least high-dose OIH, animal studies have shown that morphine-evoked nociceptive behavioral responses are not reversed by pretreatment with naloxone and one human study where results suggested that the endogenous opioid system did not appear to modulate OIH [39, 84]. The involvement of the N-methyl-d-aspartate (NMDA) receptor system is under exploration, with dose-dependent inhibition being achieved via the use of an intrathecal (IT) NMDA receptor antagonist or an NMDA ion channel blocker [85]. 

The relevance of OIH to this debate is to consider for future research studies, is there a difference in groups of patients with ORCP, those with all relevant outcome measures incorporated to ensure effective pain control of ORCP compared with a group of patients with ongoing ORCP uncontrolled, in the actual incidence of OIH. Does effective maintenance of analgesia lower incidence of OIH?

### What are the Unknowns? Pain Theories with a New Proposed ‘Clinical Overview’ for ORCP

In 1959, the sensory interaction theory Noordenbos proposed two systems of pain transmission: *‘fast with large myelinated fibers*,* slow with small unmyelinated* fibers’, in which disease that affects large fibers increases the probability of summation [86, 87]. Melzack and Wall [24], developed the *‘gate theory’* mechanism to explain when *‘pain becomes the disease’*. Mendell [88], debates the ‘gate theory’ in relation to nociceptors, described as ‘polymodal nociceptors’ by Burgess and Perl, and collaborators [89, 90] and their role in pain and sensitization is at odds with the ‘gate theory’. Trouvin and Perrot [91], described chronic nociplastic pain when pain arises from altered nociception despite no clear evidence of actual or threatened tissue damage, which causes the activation of peripheral nociceptors or evidence of disease or lesions of the somatosensory system causing pain. Dickenson noted [92], *“The Gate remains open…but there are more ways of shutting it”.* Cancer pain is complex and multidimensional, one aspect of cancer pain is selected in this paper to suggest a definition/clinical overview that may help in clinical practice and/or create a forum for debate on future research, ORCP. The pain associated with ORCP maybe ‘Functional’ [71]. Hanks et al. [93], noted, *“Morphine appears to have no clinically relevant ceiling effect to analgesia.”* This quote is referring to cancer, ORCP only. Debating the hypothesis that ORCP, treated early, with sustained effective analgesia adjusted up and down in tandem with disease trajectory may remain acute pain in nature, a ‘Clinical Overview’ in Table 3 below is suggested. This ‘Clinical Overview’ is inherently limited by the fact that it is based on a hypothesis-driven theory and is put forward as a working hypothesis only.

**Table 3:** New ‘clinical’ overview proposed for ORCP

*ORCP is opioid responsive pain*,* that initially can alert to disease*,* which may remain ‘Constant acute pain’. Pain is what the patient says it is. The opioid responsive ‘Pain’ may act as the functional antagonist to the side effects of the opioid, but requires futher research. The core issues to address keeping ORCP acute may lie in early effective pain control*,* opioid rotation to potentially eliminate tolerance plus/minus treatment of OIH*,* appropriate pain interventions*,* throughout the disease trajectory. Investigations of fMRI for some and comparisons with groups of chronic pain patients with nociplastic pain may help elucidate the hypothesis. ‘Sepsis’ and the potential for the body to produce its own analgesia with endogenous endorphin production in preparation for ‘fight-or-flight’*,* likely through neutrophil effect, requires research.*	

## Moving the Evidence Forward: “Strategies to Explore the Known into the Knowns, Unknowns” 

### Opioids and Cancer: Legal Duties and Research

Opioid analgesics are among the most prominent drug classes associated with medication incidents that have serious adverse outcomes for patients. Adverse Drug Reactions associated with opioids include respiratory depression, constipation, nausea, vomiting, abdominal pain, bloating, cramping, sedation, confusion, hallucinations, hyperalgesia, central sensitization, pruritus, analgesic tolerance, and addiction liability [94]. In the past two decades, nearly 600,000 people in the USA and Canada died from opioid overdose events [95]. As clinicians, we have both a legal and moral/ethical duty to report on opioid errors/misuse to protect patients [96–100]. The legal cases in the USA to address inappropriate promotion of opioids [98, 99], the fight for over four decades by 450 families affected by opioid issues in Gosport, UK [96, 97] and the prevention of risk-to-life from opioids [100], with *“a number of breaches of the Misuse of Drugs Act (1988) are identified in the report; this represents an especially serious finding for a unit with a necessarily high use of opiates and other controlled drugs*,*”* in the West of Ireland, (addressing the risk, led to undermining of the risk reporter, suppression of the risk), all give an overview of the complexity surrounding opioids. Since the Centers for Disease Control and Prevention (CDC) released its Guideline for Prescribing Opioids for Chronic Pain in 2016, there have been reports of misapplication for patients with pain associated with cancer [101]. Therefore, there is a fine line when dealing with opioids, with most serious adverse outcomes and facing optimum management of patients with ORCP who deserve best management.

Preclinical research, which costs approximately $28 billion annually in the USA, 50% is not reproducible; therefore, it could be classified as redundant, but all research done does add to our body of knowledge [102]. To address strategies to minimize this issue, Freedman et al. [102], emphasized the identification of a primary hypothesis and outcome measure for future research and quotes, *“the challenge of increasing reproducibility and addressing the costs associated with the lack of reproducibility in life science research is simply too important and costly to ignore.”* For cancer pain research, this paper suggests that if one component of cancer pain relief, ORCP as a primary hypothesis is re-evaluated and researched as ‘Constant Acute Pain’, ensuring a solid outcome is measurement of effective pain relief, the results could be illuminating.

Considering ‘Cluster Randomized Controlled Trials’ (CRT), where ‘groups’ are randomized, not individuals, would avoid ethical concerns of withholding opioids in patients who need them [103]. The relationships among exogenous opioids, endogenous opioids and the immune system warrant prospective large multicenter studies for cancer patients and noncancer chronic nocicplastic pain patients [91]. Such studies could provide a better understanding of the pathology of different cohorts of patients with pain.

### Opioids and Sepsis

Reviewing all major ‘Sepsis scoring systems’ in use world-wide, of note, opioids do not get a recognition of a ‘red flag’ warning pre and/or peri-sepsis [61, 62]. The incorporation of all patients on opioids into a ‘red flag’ sepsis scoring system in acute hospitals could have advantages in both keeping patients safe from opioid side effects by reducing opioid dose when necessary and aid in alerting clinicians to early warning signs of evolving sepsis. Considering that Zhang et al. [104], reported that opioid-treated patients had a significantly greater risk of death at 28 days, understanding the prodromal phase of sepsis, the potential interaction of external opioids with endogenous opioids and how to manage this phase clinically warrants further attention and controlled trials. The treatment of sepsis affects almost every specialty of medicine.

Abu at al [105], quote of Glattard et al’s [63], *“Similarly*,* others have found dramatically increased levels of endogenous morphine in the serum of patients with generalized infection in sepsis*,* severe sepsis (sepsis + end organ damage*,* hypotension*,* elevated lactate levels)*,* and septic shock (severe sepsis + refractory hypotension) compared with inflammatory states without infection such as systemic inflammatory response syndrome (SIRS).”*, reinforcing the importance of Glattard et al.’s [63] and Waldron et al.’s [60], research. The discovery of endogenous opioid peptides was in 1975. Hughes [106], Holaday and Faden [107], provided the first evidence for the role of endogenous opioid peptide involvement in the physiopathology of circulatory shock. The first suggestion for the use of naloxone in the treatment of sepsis was in 1982 [108], in 1989 [109], and other studies were outlined in the 2003 Cochrane review [35, 110, 111]. However, in the Cochrane review, all studies were published in the 1980s or early 1990s, and few papers were published since. The role of naloxone before and during sepsis warrants reactivation with further controlled clinical trials.

In the clinical setting, cancer patients with ORCP recalibrate back toward their pre-sepsis opioid dose within approximately one week of sepsis treatment [60]. In practice, reassurance patients/families that this scenario is not a ‘reaction’ to opioids but rather their own bodies’ internal preparation to fight sepsis maybe helpful. Additionally, reinforcing at presentation of opioid toxicity that patients may need to receive additional opioid, safely, after sepsis treatment, is important for patients’/families’ confidence. For patients on opioids for nocioplastic/nonopioid responsive pain, in future research, study of opioid recalibration post-sepsis treatment (elimination of tolerance) may be an effective way to assess tolerance in this patient group. This may provide a framework to further elucidate whether ORCP remains acute in nature, despite the duration of the pain. Considering the opioid crisis, Abu et al. [105], suggests *“understanding how opioids affect sepsis risk is of paramount importance.”*

### Opioids and Delirium

The clinical evidence that endorphins are likely produced pre and throughout sepsis could render a patient opioid toxic, therefore, delirious, 61% of retrospective audit (Table 2) hallucinating with other CNS side effects in the setting of sepsis [60]. With delirium throughout the trajectory of a cancer patient’s journey and at the EoL, there is understandable fear of inducing pain by lowering opioid doses; however, the side effects of opioids are clinically predictable and unpleasant. A reduction in the opioid dose, in the setting of delirium, should be considered in cancer patients receiving active effective, anticancer treatment, presumed to be septic and at the EoL, as ongoing delirium that requires antipsychotic medications may play a role in mortality [112]. This is not to suggest that all patients at EoL have sepsis, but as opioid side effects such as decreased respiratory drive, hallucinations, hyperesthesia, delirium, nausea and myoclonus are significant symptom burdens for patients; therefore, future studies incorporating opioid reduction into the methodology could be informative.

Slatkin and Rhiner [113] suggested the use of acetylcholinesterase inhibitors for opioid-induced delirium, however, this is only a case report so of limited value. Recent evidence of the effect of haloperidol on delirium during sepsis in intensive care unit (ICU) patients, in a controlled trial, is not effective, and a higher mortality rate reinforces the need for future controlled trials to reduce the use of external opioids and/or naloxone throughout sepsis [114]. Yoo et al. [115] reported that the association between opioid use and the occurrence of delirium was dose dependent, with higher doses leading to a greater risk of delirium. This study did not address whether sepsis was present or not present and/or whether opioid reduction improved delirium.

### Opioids, Cancer and Addiction

The rise above normal DA underlies the ‘high’ and addictive potential of opioids [67]. Therefore, is individualized opioid treatment appropriate for ORCP ‘normalizing’ the body’s nonimpaired lowered DA, rather than creating a rise in DA? In ORCP, is neuromodulation similar to postoperative, ICU, acute pain populations because of inflammation from active cancer, with nonimpaired DA, is it different to research done on chronic inflammation and pain of other conditions [116]? This could potentially separate ORCP from nocioplastic chronic pain, animal studies by Yung-Jen Huang, quote, *“The results suggest that GABAergic neurons drive*,* rather than inhibit*,* the development of nociceptive sensitization after spinal injury.”* [117].

Another caveat to consider reflecting on is that sometimes science identifies issues after they have been noticed in clinical practice, clinical experience had raised questions surrounding the more significant addictive potential of oxycodone versus ‘other’ regularly used opioids. Vander Weele et al. [118], later demonstrated that DA transmission within the nucleus accumbens (NAc), a key neurobiological component of motivation, is markedly different following oxycodone than after morphine sulfate delivery [118]. Morphine delivery is associated with a coincident surge in both the DA and GABA concentrations immediately following drug delivery to the NAc, which may explain why the DA concentration quickly returns to baseline levels following the delivery of Morphine, but the GABA concentration increases for longer with the addition of oxycodone [118, 119]. Comer et al., from a heroin-dependent individual, stated that “*oxycodone is the Rolls Royce of opioids”*, as it produces *“a smooth high”* [120]. DA and GABA are key to the development of addiction, and the timing of their alterations within the NAc is also relevant. This paper is referred too to elucidate what science can discover a significant time after an opioid is being used widely in clinical practice.

Gaertner et al., discuss [121], *“our frequently heard palliative care mantra neither addiction nor mortality issues are of relevance for opioid use… Addiction is always a potential concern for opioids*,* although some patients may be at higher risk than others”.* Dalal and Bruera [122], give very clear guidance to avoid addiction in cancer patients and suggest, “*“In view of the ongoing opioid overdose epidemic*,* and emerging data of Non-Medical Opioid Use (NMOU) risks in patients with cancer*,* it is of vital importance to screen all patients for their risk for opioid misuse and harm…. Through intensive education*,* ongoing monitoring*,* timely identification*,* and appropriate management of NMOU*,* health care providers can optimize the risk-to-benefit ratio to support the safe use of opioids for patients with cancer.”*

### Opioids, Application in Clinical Cancer Care

ORCP requires careful, ongoing, individualized supervision, best-case scenarios led by one specialist team throughout the disease trajectory. ORCP, once identified, should be considered as defined *“as what the patient says it is”*. All patients screened for risk of opioid abuse and harm [122, 123], alteration of the WHO step-ladder to reflect, *‘step-up*,* step-down’* in the future could potentially minimize addiction, maybe minimize tolerance and improve well-being. Evolving studies of the major neurotransmitters underlying ORCP, GABA, glutamate and DA compared with nociplastic pain are needed. Early, effective control of acute pain lessens ongoing chronic pain; [124] therefore, if ORCP is effectively controlled early and opioids are reduced as disease regresses, there may be a decreased incidence of ongoing chronic pain in cancer patients [88, 124]. van den Beuken-van et al. [125] reported that the incidence of chronic pain in cured cancer patients was 41%. The question of early, effective pain control in ORCP that may help keep ORCP acute in nature versus becoming nociplastic needs future studies. Authors suggest, *“Let’s keep ORCP acute by controlling it*,* a win-win situation.”*

As the literature is scant with evidence to support ORCP could remain acute in nature, this limits our hypothesis so other factors of relevance to the hypothesis are put forward. The need to admit a cancer patient the day before a Coeliac Plexus Block (CPB) so the patients’ opioid could be reduced with effective intervention, in anticipation of inducing opioid toxicity if reduction not done. Cochrane review [126], report, *“The mean difference for the VAS pain score at four weeks was significant *(*P = 0.004) for the experimental group (CPB). This improvement in pain control coincided with a reduction in opioid consumption; the mean difference in the use of analgesic therapy in the two groups was significantly in favor of the CPB group *(*P < 0.00001).”* Routine clinical practice involves close monitoring of opioids peri-operatively, if surgery is expected to remove a large source of the cancer patients’ pain, opioids may need reduction and if surgery not expected to remove source of pain, opioids may need increase. Hyland et al. [127], do a comprehensive review of perioperative stewardship of opioids but cancer does not get individual attention, in clinical practice, each individual case is evaluated to make best decision regards opioid reduction or not for each cancer patient based on anticipated removal of pain source or not. Analysis of Methadone used to eliminate OIH and/or Tolerance in complex cancer pain also proved a reduction in other baseline opioid post use of methadone as did Ketamine study [20, 23]. In practice if OIH and/or Tolerance meant nociplasticity the baseline opioid would not be expected to need to be reduced while still maintaining effective analgesia.

On considering further analysis of Hardy et al. [22], Ketamine study, a percentage of side effects could be attributed to toxicity of opioids. Analysis of the reduction in baseline opioids post-treatment with burst ketamine would be helpful. Of 103 of the side effects reported (cognitive disturbance 17, confusion 13, dizziness 17, hypoxia 7, somnolence 24, nausea 15 and vomiting 10) these could be attributed to opioid side effects. Based on the principle that as the proposed NMDA effect of ketamine effects enhanced analgesic relief, OIH and/or tolerance could be eliminated. Then opioid side-effects can occur due to the rendering of the baseline opioid redundant. In this study, 31 out of 93 patients suffered reactions. In comparison the oral Ketamine study of patients with non-cancer chronic pain with 60% on opioids did not have the high degree of side effects, despite a high percentage being on opioids. This could this reflect a patient group having nociplastic pain, versus the cancer population in above study having OIH and/or Tolerance without nociplasticity [21]? 

### Opioids and Respiratory Function

Dahan et al. [122], reported that 42 chronic pain patients experienced opioid induced respiratory depression (OIRD) before the year 2000 which predominantly involved morphine in cancer, but since 2000 it predominantly involved non-cancer patients. Dahan et al. [128–130], explain factors that contribute, *“reflects the increased use of opioids in patients with non-cancer pain in the last decade.”*

The scientific meaning behind Pattinson’s work [83] from almost two decades ago, also explains the recent rationale behind the Drug Enforcement Agency (DEA) *‘One Pill can Kill’* campaign in the USA, any illicit fentanyl laced on a pill the opioid naive brain has insufficient time to adjust; therefore, respiratory depression and sudden death can occur [83, 131, 132]. As one parent of the DEA videos discussed, why is the head count of people dying of unsuspected fentanyl overdose not given the same attention as COVID-19-related deaths in daily news reports [132]? The science shows that the opioid-naive brain cannot tolerate fentanyl [83]. Of course all opioids can cause OIRD as in the first three waves of the opioid crisis, fentanyl is particularly heard about the fourth wave. This reflects the combining of science intricately to the clinical reality is important, hence the rationale of this paper to hopefully initiate a debate on ORCP.

### Opioids, Cancer, Tolerance/OIH and the WHO Stepladder

As upfront this paper has concentrated on ORCP to forward a hypothesis, as many cancer pains are mixed pains, next discussion concentrates on all cancer pains.

Examination of OIH in cancer patients with ORCP versus tolerance requires further evaluation. What is the true incidence of both and what are the predictors associated with OIH occurrence? Over three decades, patients clinically presenting with tolerance appear to respond to opioid rotation as the initial step. If this step is not effective, then OIH is suspected, and the next step includes the ‘Burst Ketamine’ regime, i.e., the addition of SC ketamine to the opioid regime for a few days to cut off NMDA activity [23, 133]. Oral ketamine can also be effective [21]. plus/minus methadone [20] Tapentadol, requires further research using it for its NRI effect to access if it is opioid sparing [134–136]. 

Reducing the peripheral drive to the pain signaling system can be achieved via interventional approaches, nerve block, *i*,* e*, CPB or spinal ‘intrathecal’ opioid (IT) delivery and should be considered if appropriate [126, 137]. Ketamine is an NMDA receptor antagonist, and methadone is a mu opioid receptor (MOP) agonist and unproven yet, but presumed, with some clinical evidence, NMDA receptor antagonist [20, 21, 133]. Pregabalin is potentially dangerous since there is emerging evidence that the respiratory tolerance on long term opioids is re-set with the addition of gabapentinoids resulting in the observed increase mortality of patients on the two agents [138]. Methadone may eliminate OIH through its effect on the NMDA receptor site [139]. Tapentadol is a combination drug with the mechanism of action of stimulating inhibitory MOP and mediating noradrenaline reuptake inhibition (NRI), leading to activation of the inhibitory alpha-2 adrenoceptor at the spinal cord. It has been shown to be effective in a model of cancer-induced bone pain [71]. In a recent study on opioid-induced androgen deficiency, testosterone levels in 5/11 patients in the oxycodone group were reduced versus 2/19 in the tapentadol group [134]. It could potentially be considered as a modern-day amitriptyline with a different side effect profile, i.e., its neuropathic NRI effect from the brain down the spinal tract. By carefully selecting the rationale for tapentadol use, i.e., if OIH and/or nerve pain are suspected, and testing the immediate release form and observing whether there is a benefit [135, 136], the addition of tapentadol with a baseline MOP acting opioid for its NRI has yet to be researched.

Martyn et al. [140], explored the development of tolerance to opioids in intensive care unit (ICU) patients with acute pain, explained by ongoing inflammation in their bodies. Table 1 [140], in this paper, which presents strategies to mitigate opioid tolerance and/or OIH mirrors the messages in this paper and suggests mitigation of these problems in patients with ORCP, if effective analgesia is maintained over time, similar to ICU patients. Martyn et al. [140], quoted for ICU critical illness patients, *“Both the duration and dose affect the development of tolerance”*. Clinical practice for ORCP should involve early, individualized, patient-led, effective analgesia, opioid rotation and/or the addition of NMDA-active drugs early if required, could minimize the development of tolerance and/or OIH. Notably, the association between inflammation and the occurrence of tolerance and/or OIH in ICU in future studies could be studied in comparison to patients with ORCP to assess outcomes of tolerance and/or OIH.

Recent findings of ten times fewer opioid overdoses in cancer patients than in the general population in the USA which could suggest a difference in people with cancer versus the rest of the population [141]. We suggest that this study should be replicated in other countries, as clinical experience suggests that ‘ten times less’ is still very high for cancer patients. Lin et al. [142], reported higher levels of mood and better performance in cancer patients with pain control than in cancer patients with pain. With chronic pain, there appears to be a delay in the development of analgesic tolerance when the opioid is administered in the context of ongoing inflammatory cues that sustain the opioid analgesic response versus the absence of inflammatory cues leading to rapid analgesic tolerance [12, 140]. Cancer, by its very nature, causes ongoing inflammation until the disease responds to treatment, which could explain the difference in the development of tolerance to opioids in patients with ORCP-related nociplastic pain.

## Conclusion

Volkow and McLellan discuss areas of research needed for improved practice guidelines, including *“how to differentiate the unique properties of acute and chronic pain and how to describe the process by which acute pain transitions into chronic pain*.” [143] The new definition of *‘nociplastic pain syndromes’* for noncancer patients and those cancer patients who appear to develop nocicplastic pain versus cancer patients who do not develop nocicplastic pain should help elucidate *‘differences’* in pain types [7]. Functional magnetic resonance imaging (fMRI) studies of patients with chronic nocicplastic pain versus patients with ORCP pain could help elucidate differences in patient groups regarding the *‘acute’* versus *‘chronic’* nature of their pain.

As this is a hypothesis-driven paper, there are many obvious limitations to acknowledge, sparce data, but results above outlined show a trend that ORCP appears acute in nature. As the literature has classified cancer pain sustained over three months, there is sparce literature to challenge that ORCP may not under certain circumstances become chronic/nociplastic pain.

We believe that there is sufficient clinical bedside evidence to complement existing laboratory evidence that ORCP is acute and appears to remain acute in nature, if effective control of pain achieved early and sustained. Clinical evidence has some credibility to warrant the core of this hypothesis-driven paper being explored. The authors are fully aware of the limitations of results to back up all the debate, but the literature existing on ORCP is also still lacking as the treatment of cancer pain is suboptimal [4]. 

Can we consider opening the ‘gate’ and explore the reclassification of ORCP? Specialist Palliative Medicine/Pain Specialists should be involved in early disease in symptomatic cancer patients to ensure the continuity of care for ORCP. A debate would be helpful to educate the public of the value of early involvement of the specialty of Palliative Medicine to further reduce fears for the patient and oncologist to promptly involve the ‘Gatekeepers’ of cancer symptom control [144]. WHO recent directive: [145] *“Palliative care is most effective when considered early in the course of the illness. Early palliative care not only improves the quality of life for patients but also reduces unnecessary hospitalizations and the use of health-care services.*

Asthana et al. [13], quote, *“Though some principles of chronic pain management can be extrapolated*,* we recommend that guidelines for cancer pain management should be developed using empirical data primarily from patients with cancer who are receiving opioid therapy.”*

If indeed ORCP is ‘constant acute’ pain, then it should be controllable, albeit requiring ongoing observation and adjustment, with minimal compromise to the patient and maybe avoid development of the acute pain into nocicplastic chronic pain. A renewed overview/definition of ORCP, as ‘*cancer pain that is opioid responsive’* could create the groundwork for reevaluation of cancer pain control worldwide and/or a different prospective on how to study ORCP. (Table 3.) This paper calls for a paradigm shift in the definition of ORCP from chronic pain to consider that it might be ‘constant acute pain’. Other cancer pains are beyond the scope of this paper as this one large area of cancer pain helped elucidate this hypothesis. To quote Atul Gawande [146], *“We think our job is to ensure health and survival. But really it is larger than that. It is to enable well-being. And well-being is about the reasons one wishes to be alive.”* ORCP, alerts to disease and appears to remain opioid responsive, main message, gaining/maintaining control of the pain early to avoid pain transitioning into chronic nocicplastic pain after three months requires debate and future clinical trials to evaluate. Timing and control of ORCP maybe key to understanding how to prevent this proposed acute pain becoming chronic pain.

## Key References

2. Paice JA, Mulvey M, Bennett M, et al. AAPT Diagnostic Criteria for Chronic Cancer Pain Conditions. *J Pain*. Mar 2017;18(3):233–246. doi:10.1016/j.jpain.2016.10.020.

- This is a very comprehensive document that explores the fact that currently, no comprehensive, universally accepted cancer pain classification exists.

7. Fitzcharles M-A, Cohen SP, Clauw DJ, Littlejohn G, Usui C, Häuser W. Nociplastic pain: towards an understanding of prevalent pain conditions. *The Lancet*. 2021/05/29/ 2021;397(10289):2098–2110. doi:10.1016/S0140-6736(21)00392-5.

- This is a very comprehensive document that defines chronic non cancer pain separate to cancer pain.

20.• Hayes J, Waldron D, Levins KJ, et al. Methadone prescribed as an analgesic by a specialist palliative medicine team in an acute hospital inpatient setting: retrospective study. *BMJ Support Palliat Care*. Sep 14 2022;doi:10.1136/spcare-2022-003586.

- This is a comprehensive document that describes the clinical importance of increased endogenous morphine during sepsis.

31. Tracey I, Bushnell MC. How neuroimaging studies have challenged us to rethink: is chronic pain a disease? *J Pain*. Nov 2009;10(11):1113-20. doi:10.1016/j.jpain.2009.09.001.

- This is a comprehensive document that discusses chronic pain as the disease.

32. Volkow ND, Collins FS. The role of science in addressing the opioid crisis. *New England Journal of Medicine*. 2017;377(4):391–394.

- This is a very comprehensive document that calls out to science to assist in the Opioid Crisis.

54. Al-Hashimi M, Scott SW, Thompson JP, Lambert DG. Opioids and immune modulation: more questions than answers. *Br J Anaesth*. Jul 2013;111(1):80 − 8. doi:10.1093/bja/aet153.

- This is a comprehensive document that explores the complexity of opioids and the immune system.

63. Glattard E, Welters ID, Lavaux T, et al. Endogenous morphine levels are increased in sepsis: a partial implication of neutrophils. *PLoS One*. Jan 20 2010;5(1):e8791. doi:10.1371/journal.pone.0008791.

- This is a comprehensive document that measures endogenous morphine during sepsis.

66. Osman NI, Baghdoyan HA, Lydic R. Morphine inhibits acetylcholine release in rat prefrontal cortex when delivered systemically or by microdialysis to basal forebrain. *Anesthesiology*. Oct 2005;103(4):779 − 87. doi:10.1097/00000542-200510000-00016.

- This is a comprehensive document that unravels potential connections between delirium and excess opioids.

71. Falk S, Bannister K, Dickenson AH. Cancer pain physiology. *Br J Pain*. Nov 2014;8(4):154 − 62. doi:10.1177/2049463714545136.

- This is a very comprehensive document that explores Opioid-induced hyperanalgesia.

80. Vargas-Schaffer G. Is the WHO analgesic ladder still valid? Twenty-four years of experience. *Can Fam Physician*. Jun 2010;56(6):514-7, e202-5.

- This is a comprehensive document that is a first to suggest the WHO Step-Ladder of Pain should be reflected ‘down as well as up’.

102. Freedman LP, Cockburn IM, Simcoe TS. The Economics of Reproducibility in Preclinical Research. *PLoS Biol*. Jun 2015;13(6):e1002165. doi:10.1371/journal.pbio.1002165.

- This is a comprehensive document that questions how research is implemented.

114. Andersen-Ranberg NC, Poulsen LM, Perner A, et al. Haloperidol for the Treatment of Delirium in ICU Patients. *N Engl J Med*. Dec 29 2022;387(26):2425–2435. doi:10.1056/NEJMoa2211868.

- This is a very comprehensive document that outlines on-going delirium requiring anti-psychotic medication may play a role in mortality.

122. Dalal S, Bruera E. Pain Management for Patients With Advanced Cancer in the Opioid Epidemic Era. *Am Soc Clin Oncol Educ Book*. Jan 2019;39:24–35. doi:10.1200/EDBK_100020.

- This is a comprehensive document that has clear strategies to optimise safe Opioid use in cancer patients.

140. Martyn JAJ, Mao J, Bittner EA. Opioid Tolerance in Critical Illness. *N Engl J Med*. Jan 24 2019;380(4):365–378. doi:10.1056/NEJMra1800222.

- This is a very comprehensive document that explores treatment of acute pain in intensive care patients, that mirrors our practice.

141. • Chino FL, Kamal A, Chino JP. Opioid-associated deaths in patients with cancer: A population study of the opioid epidemic over the past 10 years. *Journal of Clinical Oncology*. 2018;36(30_suppl):230–230. doi:10.1200/JCO.2018.36.30_suppl.230.

- This is a comprehensive document that, in our view, raises the question is cancer pain the physiological antagonist to the Opioid side effects.

## Data Availability

No datasets were generated or analysed during the current study.
